# Supplementary material for: Lectin Sequence Distribution in QTLs from Rice (Oryza sativa) Suggest a Role in Morphological Traits and Stress Responses
Source: Int J Mol Sci. 2019 Jan 20;20(2):437. doi: 10.3390/ijms20020437 (PMC6359108; doi:10.3390/ijms20020437)
Supplement: Supplementary file 1 [file ijms-20-00437-s001.zip › Figure S2.docx]

B

A


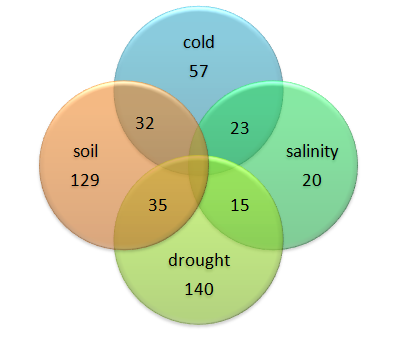

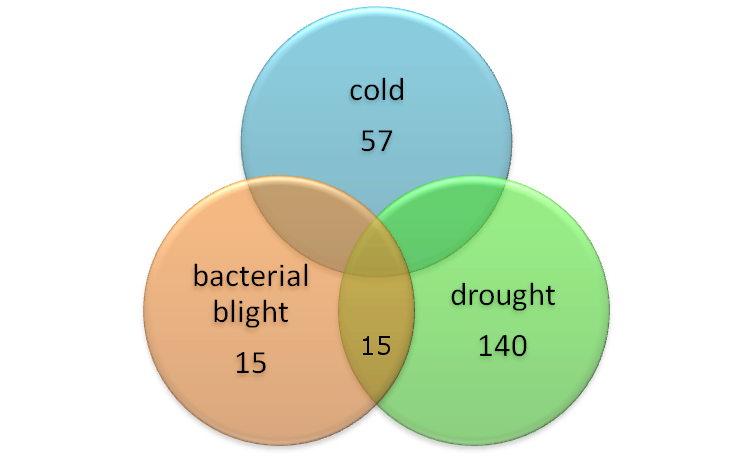


**Figure S2.** Venn diagram representing the appearance of the same lectins in more than one type of QTLs. A) abiotic stresses: cold tolerance, drought tolerance, salinity tolerance and/or soil stress. B) abiotic and biotic stresses: cold tolerance, drought tolerance and bacterial blight resistance.
