# Supplementary material for: Lectin Sequence Distribution in QTLs from Rice (Oryza sativa) Suggest a Role in Morphological Traits and Stress Responses
Source: Int J Mol Sci. 2019 Jan 20;20(2):437. doi: 10.3390/ijms20020437 (PMC6359108; doi:10.3390/ijms20020437)
Supplement: Supplementary file 1 [file ijms-20-00437-s001.zip › Table S5.docx]

**Table S5.** Overview of lectin sequences identified in *O. sativa* spp. japonica: chromosome location, distribution in QTL regions in general as well as in QTLs related to abiotic and biotic stresses.

| **Lectin Family** | # **in** **O. sativa spp. japonica, MSU IDs** | % **in** **O. sativa**  **spp. japonica, MSU IDs** | **Chromosome** | # **in** **O. sativa**  **spp. japonica RAP DB IDs** | # **in** **O. sativa**  **spp. japonica RAP DB IDs** | **Chromosome** | **% Lectins in QTLs** | **% Lectins in QTLs - abiotic stress** | **% Lectins in QTLs - biotic stress** |
| --- | --- | --- | --- | --- | --- | --- | --- | --- | --- |
| CRA | 2 | 0.61 | 4, 11 | 2 | 0.69 | 4, 11 | 100 | 100 | 0 |
| EUL | 5 | 1.52 | 1, 3, 5 | 5 | 1.72 | 1, 3, 5 | 100 | 40 | 0 |
| GNA | 134 | 40.73 | 1, 2, 3, 4, 5, 6, 7, 8, 9,10, 11, 12 | 123 | 42.41 | 1, 2, 3, 4, 5, 6, 7, 8, 9,10, 11, 12 | 93 | 66 | 17/18 |
| Hevein | 10 | 3.04 | 2, 3, 4, 5, 6 | 10 | 3.45 | 2, 3, 4, 5, 6 | 100 | 70 | 10 |
| Jacalin | 30 | 9.12 | 1, 2, 3, 4, 5, 6, 10, 11, 12 | 30 | 10.34 | 1, 2, 3, 4, 5, 6, 10, 11, 12 | 100 | 77 | 43 |
| Legume lectin | 104 | 31.61 | 1, 2, 3, 4, 5, 6, 7, 8, 9,10, 11, 12 | 81 | 27.93 | 1, 2, 3, 4, 5, 6, 7, 8, 9,10, 12 | 95 | 76 | 29 |
| LysM | 20 | 6.08 | 1, 2, 3, 6, 9, 10, 11 | 20 | 6.90 | 1, 2, 3, 6, 9, 10, 11 | 100 | 75 | 30 |
| Nictaba | 20 | 6.08 | 1, 2, 3, 4, 8, 9, 10, 12 | 19 | 6.55 | 1, 2, 3, 4, 8, 9, 10, 12 | 95 | 70 | 10/15 |
| Ricin B | 4 | 1.22 | 1,4 | 0 | - | - | 0 | 0 | 0 |
| Total # of  lectin sequences | 329 |  |  | 291 |  |  |  |  |  |
