# Supplementary material for: Lectin Sequence Distribution in QTLs from Rice (Oryza sativa) Suggest a Role in Morphological Traits and Stress Responses
Source: Int J Mol Sci. 2019 Jan 20;20(2):437. doi: 10.3390/ijms20020437 (PMC6359108; doi:10.3390/ijms20020437)
Supplement: Supplementary file 1 [file ijms-20-00437-s001.zip › Table S6.docx]

**Table S6.** Overview of total number of QTLs related with resistance or tolerance traits (according Q-TARO), number and percentage of QTLs containing lectins and distribution per lectin family. # - number, % - percentage. The percentage per lectin family was calculated as percentage according the total number of lectins in this family.

| **Number QTLs** | **Resistance or tolerance** | | | | | | | | | | | | **Others** |
| --- | --- | --- | --- | --- | --- | --- | --- | --- | --- | --- | --- | --- | --- |
|  | **Abiotic stress** | | | | | | | **Biotic stress** | | | | |  |
|  | **Cold tolerance** | **Drought tolerance** | **Lodging resistance** | **Salinity tolerance** | **Soil stress tolerance** | **Submergency tolerance** | **Other stress resistance** | **Bacterial blight resistance** | **Blast resistance** | **Insect resistance** | **Sheath blight resistance** | **Other disease resistance** |  |
| # Total | 17 | 33 | 2 | 2 | 25 | 5 | 3 | 9 | 17 | 19 | 5 | 6 | 19 |
| # Containing lectins | 10 | 22 | 1 | 2 | 19 | 5 | 2 | 3 | 7 | 7 | 4 | 2 | 11 |
| % Containing lectins | 58.8 | 66.7 | 50.0 | 100.0 | 76.0 | 100.0 | 66.7 | 33.3 | 41.2 | 36.8 | 80.0 | 33.3 | 57.9 |
| # CRA | 0 | 1 | 0 | 0 | 1 | 0 | 0 | 0 | 0 | 0 | 0 | 0 | 0 |
| % CRA | 0 | 50 | 0 | 0 | 50 | 0 | 0 | 0 | 0 | 0 | 0 | 0 | 0 |
| # EUL | 1 | 0 | 0 | 0 | 1 | 1 | 1 | 0 | 0 | 0 | 0 | 0 | 1 |
| % EUL | 20 | 0 | 0 | 0 | 20 | 20 | 20 | 0 | 0 | 0 | 0 | 0 | 20 |
| # GNA | 22 | 59 | 4 | 15 | 48 | 10 | 3 | 12 | 7 | 13 | 2 | 2 | 16 |
| % GNA | 18 | 47 | 3 | 12 | 38 | 8 | 2 | 10 | 6 | 10 | 2 | 2 | 13 |
| # HEVEIN | 2 | 1 | 0 | 0 | 6 | 0 | 1 | 0 | 0 | 1 | 1 | 0 | 1 |
| % HEVEIN | 20 | 10 | 0 | 0 | 60 | 0 | 10 | 0 | 0 | 10 | 10 | 0 | 10 |
| # JACALINS | 3 | 17 | 0 | 3 | 15 | 1 | 1 | 3 | 5 | 5 | 0 | 0 | 1 |
| % JACALINS | 10 | 57 | 0 | 10 | 50 | 3 | 3 | 10 | 17 | 17 | 0 | 0 | 3 |
| # LEGUME LECTINS | 25 | 40 | 0 | 1 | 26 | 2 | 0 | 0 | 2 | 20 | 6 | 0 | 15 |
| % LEGUME LECTINS | 30 | 48 | 0 | 1 | 31 | 2 | 0 | 0 | 2 | 24 | 7 | 0 | 18 |
| # LYSM | 0 | 14 | 0 | 1 | 9 | 0 | 0 | 0 | 4 | 0 | 2 | 0 | 5 |
| % LYSM | 0 | 70 | 0 | 5 | 45 | 0 | 0 | 0 | 20 | 0 | 10 | 0 | 25 |
| # NICTABA | 2 | 5 | 0 | 0 | 12 | 0 | 0 | 0 | 1 | 1 | 1 | 1 | 8 |
| % NICTABA | 10 | 25 | 0 | 0 | 60 | 0 | 0 | 0 | 5 | 5 | 5 | 5 | 40 |
