# Supplementary material for: Lectin Sequence Distribution in QTLs from Rice (Oryza sativa) Suggest a Role in Morphological Traits and Stress Responses
Source: Int J Mol Sci. 2019 Jan 20;20(2):437. doi: 10.3390/ijms20020437 (PMC6359108; doi:10.3390/ijms20020437)
Supplement: Supplementary file 1 [file ijms-20-00437-s001.zip › Table S8.docx]

**Table S8.** Overview of the total number of QTLs related with morphological traits and physiological traits (according Q-TARO), number and percentage of the QTLs containing lectins and distribution per lectin family. # - number, % - percentage. The percentage per lectin family was calculated as percentage according to the total number of lectins in this family.

| **Number QTLs** | **Morphological traits** | | | | | | **Physiological traits** | | | | | |
| --- | --- | --- | --- | --- | --- | --- | --- | --- | --- | --- | --- | --- |
|  | **Culm/Leaf** | **Dwarf** | **Panicle/**  **Flower** | **Root** | **Seeds** | **Shoot/**  **Seedling** | **Eating quality** | **Flowering** | **Germination/Dormancy** | **Lethality** | **Source activity** | **Sterility** |
| # Total | 24 | 19 | 27 | 27 | 33 | 16 | 22 | 16 | 9 | 5 | 23 | 22 |
| # Containing lectins | 18 | 11 | 21 | 18 | 25 | 10 | 16 | 8 | 5 | 4 | 14 | 11 |
| % Containing lectins | 75.0 | 57.9 | 77.8 | 66.7 | 75.8 | 62.5 | 72.7 | 50.0 | 55.6 | 80.0 | 60.9 | 50.0 |
| # CRA | 0 | 0 | 1 | 1 | 2 | 0 | 1 | 0 | 0 | 1 | 1 | 0 |
| % CRA | 0 | 0 | 50 | 50 | 100 | 0 | 50 | 0 | 0 | 50 | 50 | 0 |
| # EUL | 0 | 0 | 0 | 0 | 5 | 0 | 2 | 3 | 0 | 0 | 1 | 3 |
| % EUL | 0 | 0 | 0 | 0 | 100 | 0 | 40 | 60 | 0 | 0 | 20 | 60 |
| # GNA | 47 | 29 | 67 | 53 | 71 | 11 | 36 | 18 | 5 | 0 | 66 | 15 |
| % GNA | 38 | 24 | 54 | 43 | 58 | 9 | 29 | 15 | 4 | 0 | 54 | 12 |
| # HEVEIN | 3 | 3 | 6 | 2 | 7 | 3 | 5 | 0 | 0 | 0 | 4 | 1 |
| % HEVEIN | 30 | 30 | 60 | 20 | 70 | 30 | 50 | 0 | 0 | 0 | 40 | 10 |
| # JACALINS | 5 | 5 | 13 | 9 | 20 | 1 | 15 | 3 | 1 | 3 | 14 | 2 |
| % JACALINS | 17 | 17 | 43 | 30 | 67 | 3 | 50 | 10 | 3 | 10 | 47 | 7 |
| # LEGUME LECTINS | 19 | 16 | 41 | 11 | 23 | 9 | 14 | 23 | 5 | 6 | 10 | 12 |
| % LEGUME LECTINS | 23 | 20 | 51 | 14 | 28 | 11 | 17 | 28 | 6 | 7 | 12 | 15 |
| # LYSM | 4 | 4 | 9 | 9 | 10 | 2 | 7 | 3 | 1 | 0 | 8 | 4 |
| % LYSM | 19 | 19 | 43 | 43 | 48 | 10 | 33 | 14 | 5 | 0 | 38 | 19 |
| # NICTABA | 4 | 3 | 10 | 5 | 14 | 5 | 4 | 7 | 1 | 0 | 10 | 2 |
| % NICTABA | 21 | 16 | 53 | 26 | 74 | 26 | 21 | 37 | 5 | 0 | 53 | 11 |
