# Supplementary figures and images for: Lectin Sequence Distribution in QTLs from Rice (Oryza sativa) Suggest a Role in Morphological Traits and Stress Responses
Source: Int J Mol Sci. 2019 Jan 20;20(2):437. doi: 10.3390/ijms20020437 (PMC6359108; doi:10.3390/ijms20020437)

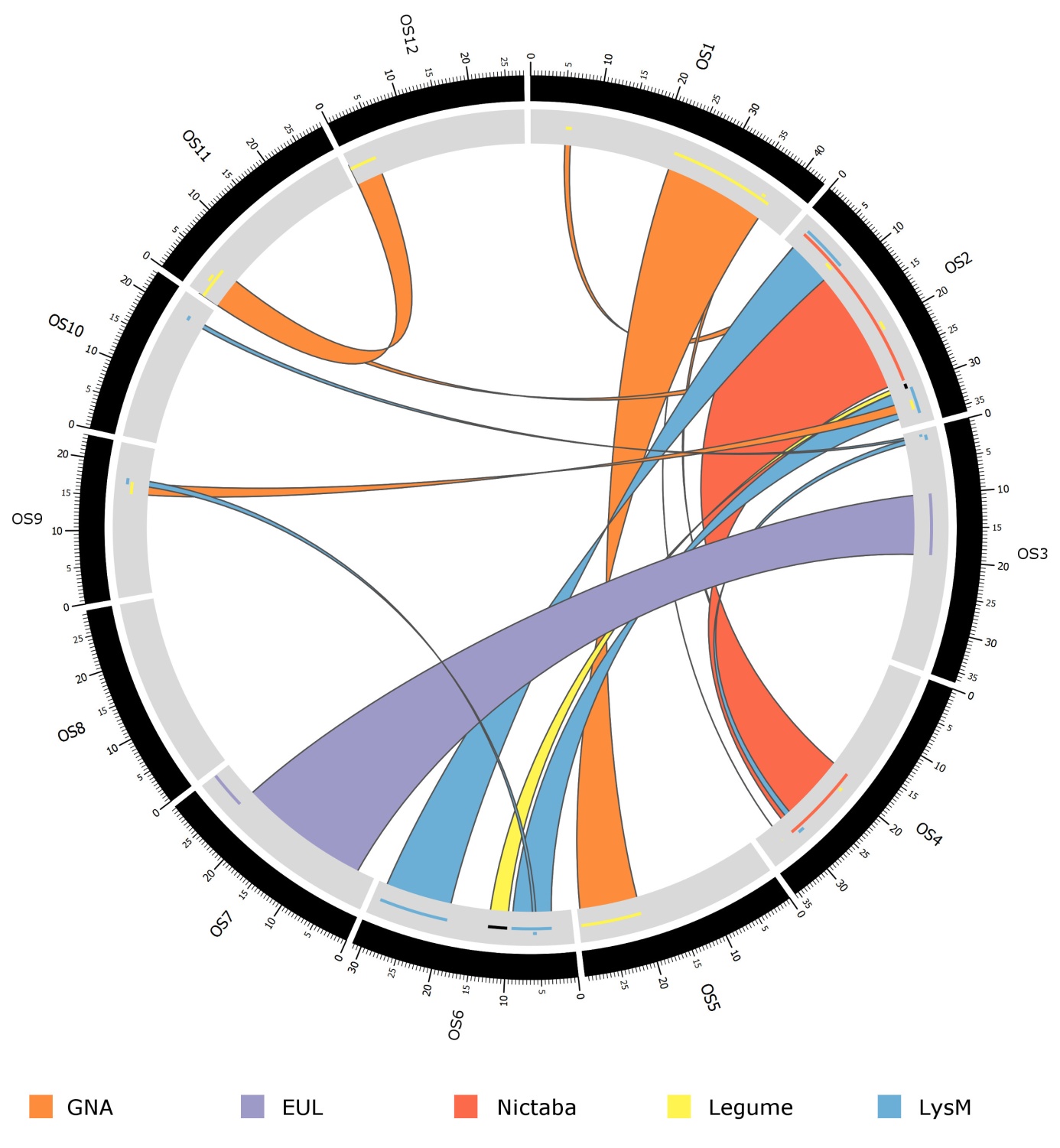


**Figure S1**. Segmental duplications in the genome of *O. sativa* spp. japonica.

Supplement: Supplementary file 1 [file ijms-20-00437-s001.zip › Figure S1.docx]
